# Investing in Healthy Brain Aging: Why Africa Must Reform Social Pension Programs

**DOI:** 10.1002/alz.095451

**Published:** 2025-01-09

**Authors:** Cyprian M Mostert

**Affiliations:** ^1^ 1 Aga Khan University, Brain and Mind Institute, 3rd Parklands Avenue, Nairobi, Kenya, Nairobi, Nairobi Kenya

## Abstract

**Background:**

African social pension schemes have irregularities. We discuss these irregularities and highlight potential reforms that may be introduced to improve healthy brain aging.

**Method:**

This analysis is based on comprehensive data from reputable sources such as the World Bank, the World Health Organisation, the United Nations, and the Organisation for Economic Co‐operation and Development. The use of such reliable data ensures the accuracy and validity of our findings.

**Result:**

We demonstrate that the current social pension scheme eligibility criteria are not designed to enforce healthy brain aging. Healthy life expectancy, which captures the number of years a person can expect to live in good health, is lower than the age for social pension eligibility criteria. For example, women’s life expectancy at birth in sub‐Saharan Africa was 66 years in 2017. However, healthy life expectancy was only 56 years, meaning that women spent four years in poor health before earning a social pension at 60 years and die 6 years later. For men, life expectancy at birth was 61 years in 2017, and healthy life expectancy was 53 years, which means men in sub‐Saharan Africa spend 12 years of their lives in poor health before earning a social pension at 65 years. Majority of these men die before earning the pension.

**Conclusion:**

Our analysis underscores the urgent need for pension reforms in Africa. We propose that pension payouts should commence from the onset of poor health in elderly citizens. The current system of delayed payouts is counterproductive to the goal of promoting healthy brain aging.

